# Barriers experienced by undergraduate students to access to mental health services: Results from a Canadian study

**DOI:** 10.1371/journal.pmen.0000109

**Published:** 2025-01-03

**Authors:** Florencia Saposnik, Dr. Mark Norman

**Affiliations:** 1 Applied Psychology and Human Development, University of Toronto, Toronto, Ontario, Canada; 2 Human Kinetics, St. Francis Xavier University, Antigonish, Nova Scotia, Canada; PLOS: Public Library of Science, UNITED KINGDOM OF GREAT BRITAIN AND NORTHERN IRELAND

## Abstract

This study examined the experiences of Canadian undergraduate students accessing mental healthcare between November 2022 to February 2023. We specifically assessed the impact of social determinants of health (i.e., gender, socioeconomic status, immigration status, English as a second language). Participants were recruited through social media platforms and by undergraduate program administrators at Canadian universities. Participants were asked to provide demographic information, answer questions about their experiences accessing mental healthcare, and to complete the mental health continuum short form (MHC-SF). Descriptive statistics and linear regression models were used to assess the association between MHC-SF and social determinants of health (e.g.: demographics, language, immigration status). Of 1098 students invited to participate, 365 participants completed the study (completion rate: 33.2%). Their mean age (SD) was 21.4 (4.6) years; 73.6% were female and 45.7% identified as non-White. Overall, the mean (SD) MHC-SF score of participants was 2.36 (0.99) out of 5. Students with low SES had lower MHC-SF scores (mean 2.08 vs 2.45; p = 0.003). The multivariable analysis showed that low SES (β -0.36; 95%CI: -0.60 to -0.12) and female gender (β -0.29; 95%CI: -0.58 to -0.012) were associated with lower MHC-SF scores. Additionally, being White was associated with higher MHC-SF scores (β -0.29; 95%CI: -0.44 to 0.54). Age, English as a second language, and immigration status were not significant predictors of mental health. High levels of stress, negative perceptions of the mental healthcare system, and limited access were the more common reported themes in the qualitative analysis. In our cohort, university students from across Canada had low MHC scores. Social determinants of health (e.g., low SES, being non-White, and identifying as a woman) were independent predictors of low MCH scores. Further studies are needed to identify specific groups at higher risk as well as strategies to overcome the suboptimal mental health among Canadian students.

## Introduction

Mental health challenges among university students have reached alarming levels in Canada, contributing to a nationwide mental health crisis [1]. Undergraduate students are particularly vulnerable to mental health issues due to the unique and multifaceted stressors they face, such as increased academic workload, significant changes in living environments, and the pressures of newfound independence [2–4]. Additionally, studies have found that students’ academic achievement can be a protective factor against mental health issues [5] however, this may be harder to achieve for first year students due to the transition into university life. Another study noted that there were increased mental health concerns during their first year at university, particularly for students entering first year during the pandemic [6]. Studies have also found that post-secondary students with pre-existing mental health conditions had worsened mental health when coupled with isolation [7]. The need for early intervention and additional programing is essential to combat this [7]. Despite this body of literature, there is a notable gap in research specifically addressing the mental health needs and experiences of university students.

Additionally, focusing on the Canadian mental healthcare system outlines structural issues to accessing care. More specifically, many studies have noted long waitlists, financial barriers, and negative experiences with mental healthcare professionals when attempting to access mental healthcare [8–10]. When reviewing the literature with regards to the listed structural issues and COVID-19, the pandemic seems to have magnified these issues [6]. Additionally, students balancing academic stressors with anxieties about their health, contributed to poor mental health [10]. Studies have also found that substance abuse was more common in the years of the pandemic due to increased stress and poor mental health [10].

Compounding this issue, social determinants of health (SDOH) significantly influence health outcomes but remain underexplored within the context of university students [11]. While there is limited research on university students’ mental health focusing on SDOH, one study demonstrates that students in the LGBTQIA2+ community experienced discrimination and exclusion from the Ontario mental healthcare system [12]. These findings speak to the need for researchers to more deeply consider how SDOH affect university students’ access to and experiences of mental health care; however, due to the limited representation from marginalized groups, we are unable to provide more fine-grained analysis of this topic in the current study. Key factors such as discrimination, immigration status, and gender have been identified as major predictors of mental health [13], yet their impact on university students’ mental health and access to care has not been sufficiently studied.

This article aims to bridge these critical gaps by investigating the experiences of undergraduate students at Canadian universities, with a specific focus on how SDOH act as barriers to accessing mental healthcare services. We examine the influence of age, gender, race, socioeconomic status (SES), English as a second language (ESL), and immigration status on students’ mental health and their ability to seek and receive appropriate care. By highlighting these issues, this study aims to inform policies and practices that can better support the mental health of this vulnerable population.

## Methods

### Participants and procedures

Data was collected from 365 participants who identified as current undergraduate students at a Canadian university. Participating universities that circulated this study included: McMaster University, University of Toronto, York University, Toronto Metropolitan University, University of Ottawa, Carleton University, Queens University, Western University, Guelph University, Dalhousie University, University of British Columbia, University of Victoria, University of Alberta, McGill University, University of Waterloo, Laurier University, University of Calgary, Brock University, University of Winnipeg, University of Windsor, Brandon University, and the University of Saskatchewan. The survey was circulated by department administrators from the universities listed above. More specifically, the departments contacted depended on what emails were easily accessible online, however, the majority were social sciences and science programs. As a result of this sampling, some programs (i.e., health sciences, nursing, engineering) were underrepresented and, therefore, the study is not representative of the entire Canadian undergraduate population. Participants were also recruited through crowdsourcing as the survey information was also posted by students on multiple social media platforms (Snapchat, Instagram, LinkedIn, and Facebook). These specific social media platforms were chosen as they are amongst the most popular for young adults. However, this recruitment strategy may have limited some students from participating due to issues with access to digital technologies and internet availability.

Data was collected between November 2022 and February of 2023. The survey automatically removed participants who answered “no” to “Are you a current undergraduate student at a Canadian university?” and removed anyone who failed the attention check. The attention check in the survey stated: “Please indicate "strongly agree" for this question to show that you are paying attention.” It included the following 5 response options: Strongly agree, Agree, Neutral, Disagree, Strongly disagree. Those who failed this check, were not current undergraduate students, and/or did not fully complete the questionnaire were removed from the sample. We included the attention check because the survey took approximately 10–20 minutes to be completed; given the length of the survey, we felt that including an attention check mid-way was essential to ensure participants were not skewing results by clicking options randomly.

### Ethics statement

The study was anonymous. Participants received no compensation and had the liberty to drop from the study at any time. Data collection occurred online through Lime Survey (https://www.limesurvey.org/).

This study received ethics approval from the McMaster Research Ethics Board (approval number: MREB #6148). The letter of information and consent preamble form was provided prior to the survey questions. Participants needed to click the option “I agree” to proceed to the questionnaire.

### Measures

Within the survey, participants answered demographic questions about their age, gender identity, race, SES, immigration status, and English as a second language (ESL) status. SES was self-reported as lower class, lower middle class, middle class, upper middle class, and upper class. Participants also completed the mental health continuum short form (MHC-SF) scale [14] which measures the mental health of participants over the past month (e.g., “During the past month, how often did you feel happy”, ∞-Cronbach = 0.908). This scale includes 14 items that can be rated on a 6-point Likert scale (0 = never, 5 = everyday). The test focuses on a variety of aspects of mental health including psychological, social, and emotional well-being [14]. Each participant is then scored out of 70 and can also be scored from the subcategories if needed [14]. Participants were also asked to respond to 5 open-ended questions about their experiences accessing mental healthcare. More specifically, they were asked the following prompts:

1. In the space below, please describe your experience with your mental health this past year.
2. In the space below, describe your experience accessing support for your mental health (this could be informal support, formal support, or how you dealt with your mental health if you did not seek support).
3. If you attempted to access mental healthcare (either formal and/or informal), please describe whether you experienced any barriers in accessing care, and if this further impacted your mental health (please put N/A if this does not apply to you).
4. If you did experience any barriers, why do you think this was?
5. How did your experience in accessing support further impact your mental health?

### Statistical analysis

Descriptive statistics were used to summarize the main results. To analyze factors associated with MHC-SF, we built linear regression models. Multivariable analysis was adjusted for age, gender, SES, immigration, race, and ESL status. Self-reported SES was categorized into low and medium-high (self-reported as lower lower-middle, middle, upper-middle, and upper). We tested linearity and conducted a sensitivity analysis by including race and the two main categories (White, Asian) to determine the consistency of our estimates (e.g., β coefficients and 95%CI). Potential multicollinearity issues were assessed using Pearson correlations and variance inflation factor (VIF). We considered values below 5 as being low collinearity [15]. There was a low correlation among the social determinants of health we examined (specifically, gender, English language learners, immigration status, SES, and race).

The impact of SDOH of Canadian undergraduate students’ experiences when attempting to access mental healthcare was assessed in a qualitative analysis through social representations theory (SRT). This theory attempts to understand participants’ thoughts on a key issue rather than focusing on the accuracy of their viewpoints [16]. More specifically, we focused on participants perceived mental health and barriers to accessing treatment or resources, rather than the accuracy of it. An intersectional analysis was used to analyze qualitative data. The responses to the five qualitative questions were analyzed through thematic analysis, a methodological approach which focuses on patterns within the responses and outlines the major themes within them [17].

## Results

Of 1098 participants, 365 participants completed the study (completion rate 33.2%). Overall, the mean age (SD) was 21.4 (4.6) years old; 73.6% were female, 40.5% self-identified as middle SES, 197 (54.3%) of participants were White, whereas the remaining 45.7% identified as part of racial minority groups. English was the first language among 80.8% students; 75.1% identified as non-immigrants and 23.1% had an immigrant status. The MHC-SF had 14 prompts, which were then combined into a mean score for each participant (refer to Table 1 for further sample characteristics).

**Table 1:** Main characteristics of participants.

Characteristics	N = 365
	n (%)
Age, years, mean (SD)	21.4 (4.6)
Sex, female, n (%) Sex, male, n (%) Self-identified gender minority, n (%)	269 (73.6) 60 (16.4) 36 (9.9)
Race: White Asian Mixed/Biracial Black Indigenous/Métis Other/self-identified SES: Low Lower-middle Middle Upper-middle Upper Prefer not to answer SES Immigrant status English as first language, %	198 (54.3) 53 (14.5) 17 (4.5) 10 (2.7) 4 (1.2) 83 (22.8) 28 (7.7) 73 (20) 148 (40.5) 101 (27.7) 10 (2.7) 5 (1.4) 84 (23.1) 295 (80.8)
English as secondary language, %	70 (19.2)
MHF-SF score, mean (SD)	2.36 (0.99)

Numbers between brackets represent percentages, unless otherwise specified.

MHC-SF: Mental health continuum short form (MHC-SF) [14].

### Quantitative

The MHC-SF scores were then averaged from all participants and led to a mean score of 2.36 out of 5, concluding moderate-low mental well-being of participants. More specifically, lower mental health scores were associated with lower SES (compared to their counterparts (2.06 vs. 2.45; p = 0.003) and being non-White (p = 0.021).

**Fig 1:**
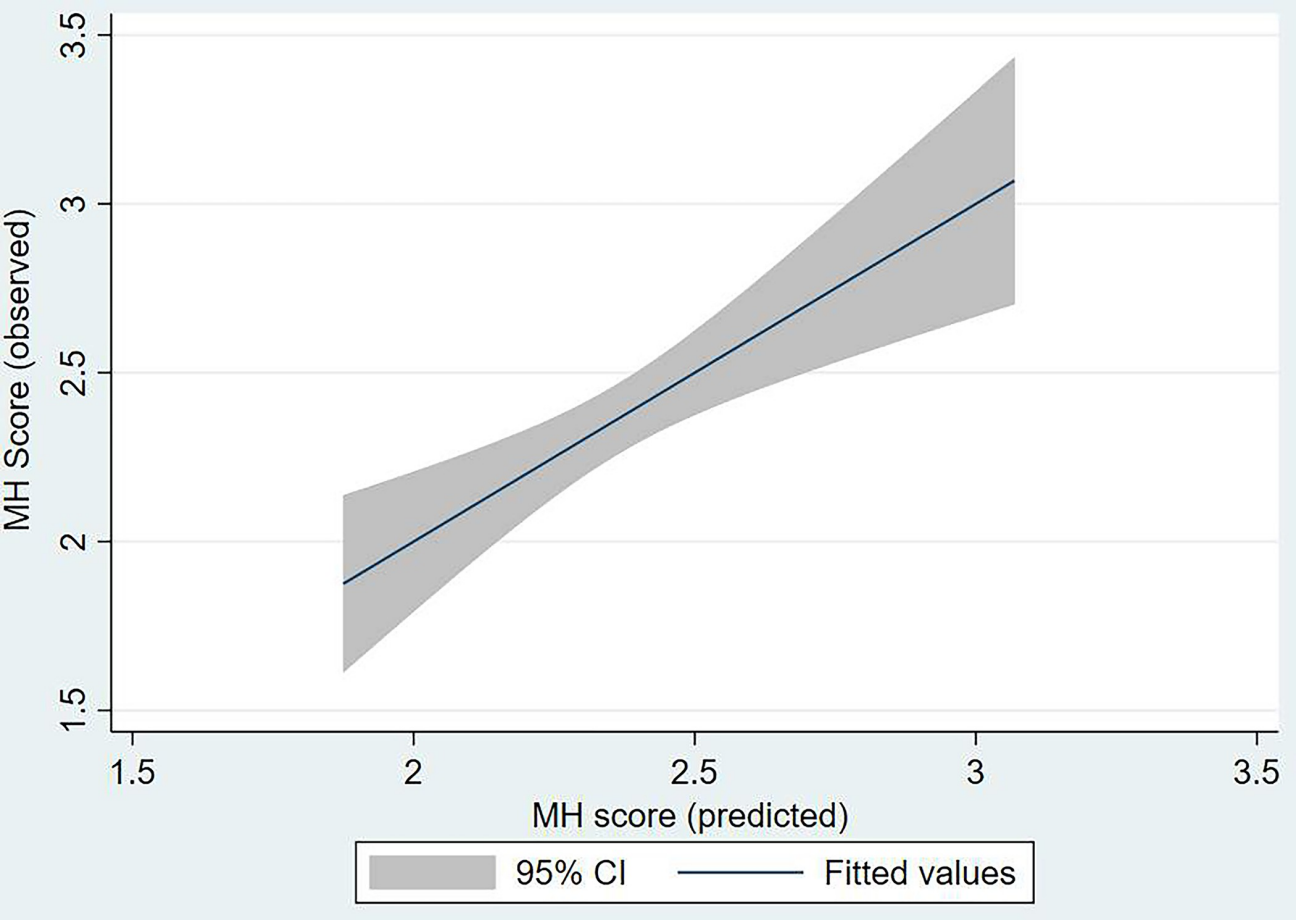
Observed vs. predicted probability of MHC score by linear regression model.

In multivariable analysis, low SES (β -0.36; 95%CI: -0.60 to -0.12), and being female (β -0.29; 95%CI: -0.58 to -0.012) were associated with lower mental health scores (Table 2, Fig 1). Additionally, identifying as White was associated with a higher mental health score (β -0.29; 95%CI: -0.44 to 0.54). Our results were consistent when race was added into the analysis. White participants had significant higher mental health scores when adjusted for covariates (Table 2). Identifying as Asian was not associated with lower mental health scores in the multivariable analysis (Table 2).

**Table 2:** Factors associated with mental health status.

	Model 1 (without race)		Model 2 (including White vs. Minoritized race)		Model 3 (including all racial groups)	
Predictors	Beta	95% CI	Beta	95% CI	Beta	95% CI
Age	0.015	-0.013 0.042	0.020	-0.007 0.048	0.017	-0.011 0.044
Gender (woman)	-0.29*	-0.58–0.01	-0.34*	-0.64–0.04	-0.35*	-0.65–0.04
Low SES	-0.36†	-0.60–0.12	-0.40†	-0.66–0.15	-0.38†	-0.64–0.13
ESL	-0.084	-0.39 0.22	-0.045	-0.27 0.36	-0.059	-0.27 0.38
Immigration Status	-0.073	-0.36 0.21	-0.094	-0.22 0.41	-0.06	-0.29 0.31
Race	--	--	0.29*	0.044 0.54	- 0.25	-0.53 0.032

Age as dichotomous variable; age by median split.

* p <0.05

† P<0.01; SES: Socioeconomic status stratified as Low vs. mid/high.

**Fig 2:**
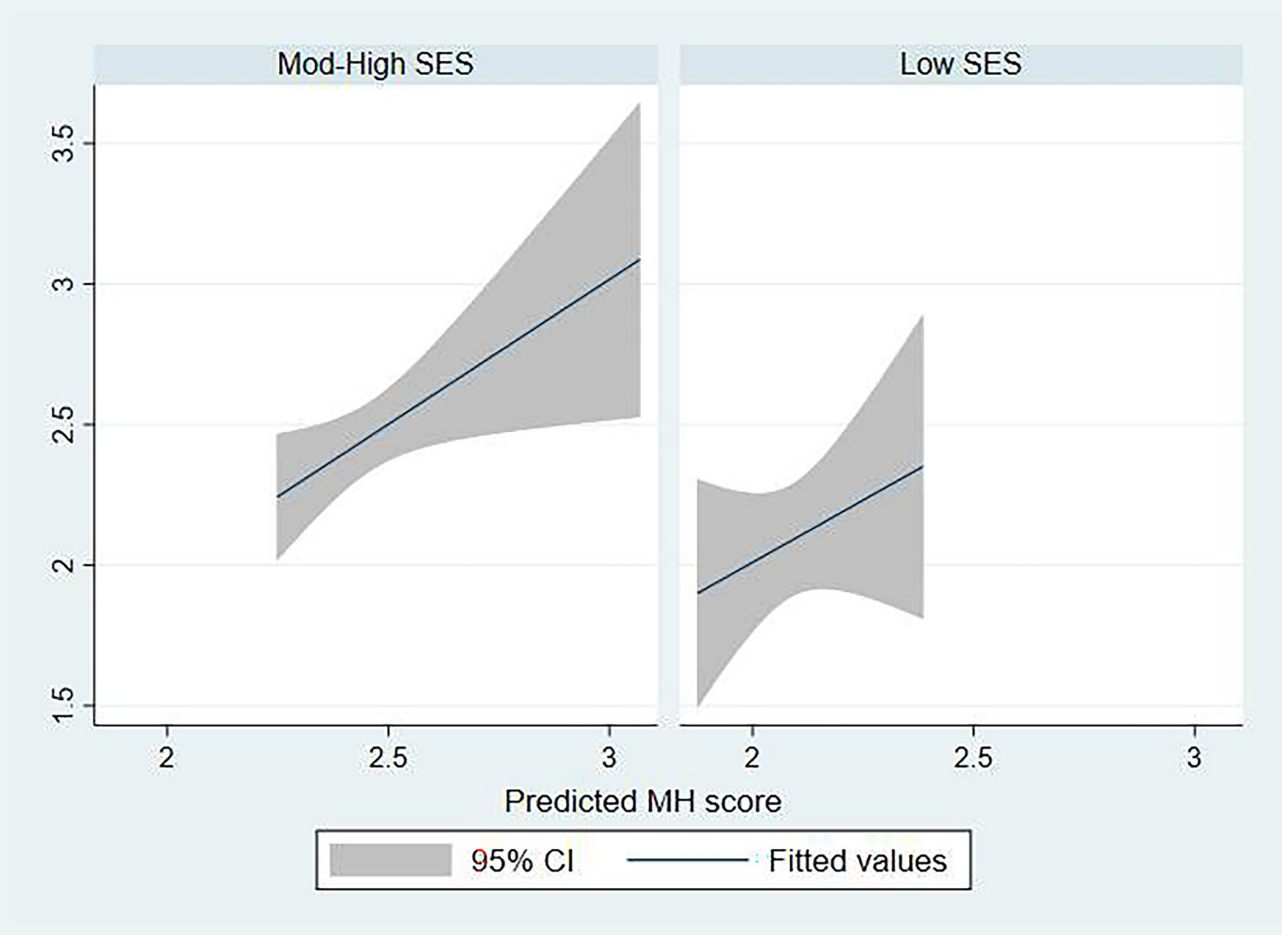
Observed vs. predicted MHC score stratified by SES.

Fig 1 outlines the linear relationship between the observed vs. predicted mental health scores of participants. Additionally, Fig 2 represents participants’ mental health scores stratified by SES. Age, ESL, and immigration status were not predictors of accessibility to mental health care.

There was a low correlation among the social determinants of health we examined (specifically, gender, English language learners, immigration status, SES, and race). Multivariable regression models showed low VIF with a mean of 1.17 and all social determinants of health had values below 1.5, suggesting no multicollinearity [15].

### Qualitative findings

Our qualitative responses were coded using thematic analysis. Table 3 outlines the themes and subthemes that arose in students’ responses to the qualitative prompts 1–5 (outlined above).

**Table 3:** Themes and subthemes from qualitative prompts 1–5.

Themes	Subthemes
1. Academic Stress	• Adapting to courses • Adapting to university life
2. Poor mental health	• Diagnosis/symptoms • Isolation • Hopelessness
3. Negative perception of mental healthcare	• Clinicians lack understanding • Isolation
4. Lack of accessibility	• Expensive • Uncertainty • Low quality • Long waitlists • Limited appointments
5. Accessing informal support	• Alternative to formal support • Increased accessibility
6. Mental health positively impacted (informal support)	• Change in perception • Increased feelings of ease

### Stress

For the theme of stress, many participants explained that adapting to in-person courses after the pandemic was difficult. More specifically, participants noted that they felt unprepared for the changes that come with in-person learning. One participant explained, “The stress of school this year has caused me to struggle with my mental health. Being back in person has added additional stress.” As illustrated by this statement, Canadian students experienced a significant amount of stress due to returning to in-person learning.

Participants also noted that they felt overwhelmed with adapting to university life (i.e., living away from home, preparing meals, etc.). One participant explained, “I’ve been experiencing the stress of moving from home, dorm life, leaving my friends, adjusting to university workload from a much lighter high school workload, and feeling quite lonely in the process.” Additionally, some students explained that the stress of university coupled with the changes of living away from home impacted their mental health.

### Poor mental health

Poor mental health was another common theme and was typically described by students using words such as “poor”, “struggling”, and “difficult”. Many students explained that they felt quite isolated due to poor mental health. Some felt as though they were alone in their struggles and as though their mental illness would stop them from socializing. Overall, students’ mental illness was both a source of social isolation and a barrier to combatting feelings of loneliness.

Many participants also expressed feelings of helplessness and/or hopelessness when they faced barriers to mental health treatment, such as long waitlists or low quality of care. One student wrote: “The experience I had with the nurse practitioner and the office’s (absent) psychiatrist was detrimental to my mental health. The fourth time the psychiatrist cancelled, I had to go home from my job because I was inconsolable and hopeless.” Another student explained,

The services I received made it feel like there was really nothing to be done to help my mental health and the difficulty of accessing the supports discouraged me from trying again when I suspected the care I’d receive anyway after going through the process would not be helpful…

Overall, as indicated by these quotations, students’ mental health was negatively impacted due to the quality of care they received, thus leaving them feeling hopeless.

### Negative perception of healthcare system

Many participants also held a negative perception of both the public and university healthcare systems. For example, many respondents felt as though their physicians did not understand their experiences and, in turn, felt quite helpless leaving their appointments. More specifically, the lack of connection with practitioners left participants questioning the competency of the clinician. One student noted, “I’ve tried talking to the therapists at [university redacted] though I don’t think they are equipped to handle my situation. I’m trans and have already completed any medical transition that I want, when I go to therapists and want to speak about the micro aggressions I face as a trans person, they don’t know how to respond. They’re focused on the medical side of things.” As seen through this quote, some participants felt more helpless given that clinicians were not attentive to their lived experiences, including intersecting social determinants of health such as being a transgender student.

Many participants also explained that accessing support felt very isolating. Some thought this experience was due to the stigma surrounding mental health, whereas others thought it was due to the mental healthcare systems they used being ill-prepared to provide services to so many students. A participant went on to explain,

Outside of the school, I found accessing mental health support quite easy, although very expensive. Within the school, I find a lot of mental health help to be online and do-it-yourself. For three years of my undergraduate degree, I was unaware of nearly all of the services my school has to offer in mental health. They were never advertised during orientation, and once found, the waiting time for an appointment is crazy.

Echoing the sentiments of the quoted participants, other students used the word “difficult” to describe their experiences within the university mental healthcare. Overall, attempting to access care and being met with a variety of barriers (later outlined) made students feel discouraged and alone in their struggles.

### Lack of accessibility

Another common theme was the lack of accessibility when attempting to access mental healthcare. Many participants explained that due to the cost of mental healthcare, they were unable to continue treatment. One participant explained,

I did see a therapist once, but the cost is too expensive, ~100$ per hour. My university insurance only covers ~[$]800 in therapy. I know that only ~8 sessions won’t be enough to help me, so I decided to see a councillor [sic] at the university, but even though I appreciate the councillor’s [sic] time, she lacks the skills to provide therapy.

Like this participant, many students outlined that they could not afford therapy themselves and did not have insurance to cover the number of sessions they needed, which deterred some from starting therapy altogether. Thus, financial strain is a large contributor to the willingness of students to seek out support and is a large barrier to continuing therapy.

Another key issue many participants noted was the uncertainty that came with mental healthcare; many did not know where to access support either through their university or through the provincial healthcare system. One participant noted, “I found it difficult to know where to turn to get support. The first person I spoke out to was my GP at the [university redacted] Wellness Centre, because I was at such a loss of what to do. I knew what kind of help I needed, but didn’t know how to access it and access it properly.” Along with the uncertainty of seeking support, navigating the mental healthcare system was also noted as being quite difficult.

Additionally, many students explained that they were deterred from seeking formal support for their mental health due to the long waitlists they experienced. Many students noted that they felt like their school’s mental health system was not able to support the increasing need from students to receive mental health support. One student explained,

The whole process of seeking help on campus was long and tedious and it didn’t matter [i]n the end as I was unable to receive help anyways. Not having a vehicle made finding help off campus difficult and I wasn’t comfortable getting help online. My school couldn’t accommodate all of the people seeking help.

Similar to this participant, many students described the waitlists using phrases such as “extremely long” and “too long”. Moreover, these quotes exemplify the negative perception that comes with long waitlists as many students attempting to receive care were hoping to receive it much sooner. In conjunction with having long waitlists to begin receiving treatment, students also noted that once they were in the system, they needed to wait very long between appointments. Many felt that their mental health would be better if they had been able to access more frequent appointments. Thus, limited appointments also impacted students’ well-being and there is a large need for improving this aspect of care.

### Accessing informal support

Due to the issues stated above, many students viewed informal support as an alternative to formal treatment. One student noted, “The support I sought was not from the formal mental health doctor, but only from my parents. We have voice and video chat every weekend, we talked about our lives and encouraged me to continue the study, and this gave me much strength on continuing my school work.” Additionally, many students mentioned relying upon “friends”, “family”, and self-care activities such as walks, exercise, and journaling, to cope with mental health struggles. These statements outline the alternative forms of support students used while facing barriers in seeking formal support.

Participants also noted that informal support was easily accessible due to being able to seek support from friends and family. One participant wrote, “I have not sought out formal support. I have however discussed with some trusted family and friends within my inner circle. In instances where I felt myself being worked up, my family and friends were able to talk me through it as well as offer some suggestions for dealing with the issue at hand…” As exemplified by this quote, students often have family and friends more readily available through technology that allows them to access informal support with much more ease than formal support.

### Mental health positively impacted (informal support)

For students who sought out informal support, many noted positive effects on their mental health. Some felt that talking to friends and/or family provided them with a new perspective on their issues and allowed them to feel more positive afterwards. One participant explained, “Talking and receiving support from my friends and family had a huge positive impact in my perception and outlook on situations/issues I struggled with.” Participants also used the word “improved” when describing their mental health after accessing informal support. These quotes show the value of informal support in helping students.

Students also felt more content and relaxed when accessing informal support, possibly due to the avoidance of many barriers explained in the above prompts. One student explained, “I access support through family and friends, they impact my mental health by being able to be present and attentive. Makes me feel a little bit at ease compared to before.” This quote exemplifies that students accessing informal support allowed them to share their feelings and as a result made them feel better.

## Discussion

By exploring undergraduate students’ mental care access in Canada, this study uncovers barriers and realities faced within the Canadian and university healthcare systems. By focusing on SDOH, we identify how these as critical factors shape and often hinder students’ accessibility to mental healthcare.

We found that a lower SES and being a woman were associated with 36% and 29% lower mental health scores, respectively. Our study also found that many students experienced poor mental health, negative perceptions, and experiences within mental healthcare systems both in university and formal healthcare sectors, which aligned with our quantitative results of relatively poor mean mental health scores and the theme of poor mental health. The lack of accessibility and poor experiences within the mental healthcare system as outlined in the qualitative responses further supported the low mean mental health score. In our quantitative findings, lower SES was a predictor of poor mental health which informed our qualitative analysis where students also noted that they used informal support to mitigate the lack of accessible formal support. Our qualitative themes also included many students noting the importance of informal support through friends and family; given that many participants were Canadian, the large access to informal supports due to proximity were clear.

Our results are aligned with previous reports showing the role of stressors on student’s mental health [2,3,18]. The finding that SES played a large role in students’ ability to access mental healthcare also aligns with a previous Canadian study, which found that postsecondary students perceived the cost of mental healthcare to be a primary deterrent to seeking formal support [19]. Like Samuel and Kamentetsky (2022) [19], our study found that many students relied on informal mental health supports in lieu of formal care. Interestingly, our findings revealed that such behaviour persisted even when cost was not a barrier to accessing treatment (i.e., students attempting to access care through their university or having parents willing to pay), primarily due to long waitlists for care. These barriers were also identified in pre-pandemic studies [20], suggesting broader structural factors in the Canadian healthcare system affecting university students’ willingness or ability to access formal mental healthcare.

Scholars recognize the need for greater attention to how SDOH intersect to produce unequal health outcomes for diverse populations (e.g., [21]). While we were not able to produce a fully intersectional analysis in the current study (see limitations discussed below), our findings do point to some ways in which various SDOH combine with university student status to shape perceptions of mental health and experiences of accessing care. Quantitative findings found that being white was associated with higher mental health scores, while being low-SES and female were associated with lower scores. Qualitative findings showed that, despite predominantly identifying as middle class, many participants clearly identified financial barriers to accessing mental health care. Further, although individual participant narratives point to the challenges that can be faced by people in marginalized social positions (e.g., transgender, low-income) when attempting to access high quality and dignified mental health treatment. Other factors, such as immigration status and being an English language learner, could have also intersected with other SDOH to shape mental health experiences, however, the small percentage of participants in these categories (23.1% and 19.2%, respectively) meant we could not determine whether these factors compounded. Overall, further research is needed to examine the direct correlations between intersecting social determinants of health and their impacts on health and access to healthcare.

Some limitations to note include the diversity of our research sample. The majority of our sample identified as female, (73.6%), white (54.3%), middle class (40.5%), and having English as their first language (80.8%). As such, the findings are limited in their ability to provide insight into marginalized groups facing challenges related to SDOH, such as racialized individuals, English language learners, gender non-conforming students, and those with lower socioeconomic statuses. While individuals identifying with these groups were included in the study, they were not widely represented and we cannot speak to their unique experiences. However, given that social marginalization can negatively affect health outcomes, we can speculate that student populations who face intersecting forms of oppression (e.g., racism, homophobia, transphobia, etc.) may report even worse mental health outcomes and challenges accessing mental health care—an area demanding future research. Furthermore, the effects of culture on the understanding of mental health will vary throughout different countries. This, coupled with the various healthcare systems in the world make our findings nonrepresentative for post-secondary students around the world. However, the need for better mental healthcare supports seems to be relevant in various contexts and can inform changes in educational settings as we will further outline in our recommendations.

Despite these limitations, our findings highlight the accessibility constraints to mental health care services, and some barriers related to SDOH (e.g., SES). Our findings underscore the urgent need for targeted research and interventions addressing the unique mental health challenges faced by university students, shaped by their diverse social determinants of health.

## Recommendations

Based on our findings, we believe that policy changes are essential to combat the public health concern of poor mental health and access to care. Some policy changes include a revision to the Canada Health Act to include mental healthcare an essential form of care. As for programming, the government could provide funding to support individuals who may be facing financial barriers to access mental healthcare. These programs could be through organizations subsidizing mental health support or linked to individual’s health cards. The government should also be considering those who are uninsured which provides an additional financial barrier when seeking care. Additionally, increasing funding for telehealth to combat the issues of large waitlists and wait times could allow for more Canadians to receive support.

Other practices that would help combat some issues discussed by participants would be for universities to increase their acceptance rates to counselling psychology and related programs to have a larger output of people who can provide mental healthcare. Universities should also focus on increasing their funding towards their university healthcare system; specifically at the student wellness centres that provide mental health support. By improving the infrastructure and employing more mental health professionals’ students would not be faced with the long wait times and waitlists. Universities could also offer a sort of payment plan or subsidizing program for students to be able to access mental healthcare through the institution as many providers within the institution keep similar prices to those who can be found outside of the university. Lastly, students noted negative experiences with some mental healthcare professionals; focusing on screening to avoid practitioners who may not be a good fit for students is essential.

Lastly, both universities and the Canadian government should prioritize providing information regarding how to seek and access mental healthcare. The theme of confusion and feeling overwhelmed by the system caused many students to avoid seeking care. Providing clear and easily accessible information, including in multiple languages that reflect the diversity of university campuses, will be useful for everyone. Additionally, having sections specific to those who may be faced with unique situations such as not being a current citizen, being an English language learner, etc. would be beneficial.

## Conclusion

Canada as well as many other countries, faces a significant mental health crisis [22]. Our study addresses a gap in the existing literature and provides the foundation for conducting similar studies in other countries. Furthermore, we identified the challenges imposed by limited access to mental care services as reflected on lower MHC scores among university students. Costs, out-of-pocket expenses, extended wait times for appointments, and insufficient clinician training, would act as deterrents to seeking care. Therefore, addressing these issues through increased funding for the public Canadian mental healthcare system and university-based mental health services is crucial to improving the quality of care and encouraging continued support-seeking among students.

Future research would benefit from including diverse samples that would be more representative of the general population, as this could further inform policy. Additionally, studies focusing on minoritized communities (i.e., English language learners, immigrants and refugees, gender non-conforming individuals, racialized individuals etc.), would help outline the unique barriers faced by these groups and may allow for the implementation of support programs specific to their group. Lastly, expanding mental health research to other areas of the world where culture informs the understandings of mental health and illness would be essential to then provide actionable forms of support.
